# Surgical prognosis in epiretinal membrane: 12-month outcomes after membrane peeling in a 5-year cohort with OCT biomarkers

**DOI:** 10.1186/s40942-026-00853-y

**Published:** 2026-04-21

**Authors:** Piero Barrera-Arshavin, Álvaro Bofill-Ramirez, Antonia Bayo-Burgos, Eduardo Urrejola-Irarrazabal, Josefina Camelio-Opazo, Juan Ignacio Verdaguer-Diaz, María José Rivas-Figueroa, Luis Filsecker-López, José Manuel López-Astaburuaga, Sergio Zacharías-Santamaría, Jorge Orellana-Rios, Efraín Pérez-Argandoña

**Affiliations:** 1 Fundación Oftalmológica Los Andes (FOLA), Santiago, Chile; 2 University of Los Andes, Santiago, Chile; 3 Department of Ophthalmology, Fundación Oftalmológica Los Andes (FOLA), Santiago, Chile

**Keywords:** Epiretinal membrane, Vitrectomy, Optical coherence tomography, Biomarkers, Visual acuity

## Abstract

**Background:**

To determine the functional and anatomical outcomes at 12 months after pars plana vitrectomy (PPV) for epiretinal membrane (ERM) and the independent prognostic value of preoperative biomarkers in optical coherence tomography (OCT).

**Methods:**

Five-year analytical study of 340 eyes with ERM in stages (Govetto G2–4). Best-corrected visual acuity (BCVA) logMAR and OCT were evaluated at baseline, 1, 3, 6, and 12 months. The primary objective was to determine the change in BCVA at 12 months (ΔlogMAR 12 m – baseline). Univariate tests, ANCOVA, longitudinal mixed linear models, and logistic regression were applied for gain ≥ 0.2 logMAR. The models were adjusted for baseline VA, age, sex, aetiology, surgical technique, and analysis of biomarkers in OCT.

**Results:**

Baseline VA was 0.54 ± 0.44 logMAR (approximate Snellen equivalent: 20/70); distribution according to G2 (*n* = 166), G3 (*n* = 146), G4 (*n* = 28). VA improved significantly at 12 months (Wilcoxon *p* < 0.001), equivalent to 11–12 ETDRS letters. Secondary ERMs showed greater unadjusted gain than idiopathic ones, but aetiology was not an independent predictor after adjustment. The surgical technique was not independently associated with VA at 12 months (β=+0.031 logMAR; 95% CI; *p* = 0.408) or with responder status (OR 1.18; 95% CI; *p* = 0.625). By severity, advanced stages had worse baseline visual acuity (VA) and greater absolute gains (Kruskal–Wallis H = 7.61; *p* = 0.0223), although with lower final VA compared to lesser stages. Baseline VA was the dominant predictor in all models (β=−0.766; 95% CI; *p* < 0.001). Among preoperative biomarkers, COST line disruption independently increased the probability of response (OR = 2.08; 95% CI; *p* = 0.013). DRIL, EZ disruption, and central bouquet alterations did not reach significance after adjustment. Mixed models confirmed early improvement, maintained up to 12 months. Kaplan–Meier showed faster time to response in advanced stages.

**Conclusions:**

PPV for ERM achieves significant improvement at 12 months. Baseline VA is the main determinant of prognosis. According to Govetto’s classification, the greatest gain occurs in advanced stages, but with lower final VA. The COST line emerges as the only preoperative biomarker with independent prognostic value for achieving ≥ 0.2 logMAR; the others provide limited utility when baseline VA is considered.

**Trial registration:**

Not applicable.

## Introduction

Epiretinal membrane (ERM) is a common macular pathology characterised by avascular fibrocellular proliferation on the surface of the internal limiting membrane (ILM) [1]. In most cases, it may be idiopathic or secondary to various pathologies, such as diabetic retinopathy (DR), retinal vascular occlusions (RVO), uveitis, trauma, retinal detachment (RD) or following cataract surgery [2, 3].

Idiopathic ERM usually occur in older adults and are a major cause of visual impairment in this age group [4], with posterior vitreous detachment (PVD) playing a major pathogenic role, as reported in most series [5].

Epidemiological studies have reported an overall prevalence of ERM of 6–11%, increasing with age to 15% in people over 70 years of age [4–6]. Clinically, patients report decreased visual acuity (VA), metamorphopsia, aniseikonia and, less frequently, monocular diplopia or binocular interference, with an impact on visual function and quality of life [7, 8].

OCT has optimised diagnosis, improving the stratification and follow-up of ERM [9]. Structurally, it is visualised as a hyperreflective band over the ILM, with associated signs of traction such as radial folds, increased central macular thickness (CMT), cystoid macular oedema and alterations in the outer layers, disruption of the ellipsoid zone (EZ) [10–12]. In 2017, Govetto et al. [13] proposed an OCT classification into four stages (G1 to G4) based on the loss of foveal depression and the presence of the ectopic internal foveal layer (EIFL), a structure that reflects a reorganization and establishment of a bridge of the inner layers over the fovea [14]. This correlates with clinical and anatomical findings, which in different series describe worse VA as the stage increases [15, 16].

As a result, various structural biomarkers with prognostic value in ERM surgery have been proposed, such as CMT, EZ integrity, central bouquet traction of the outer retina (OR) manifested by the most characteristic sign of cotton ball, and the presence of disorganisation of the inner retinal layers (DRIL) [17, 18].

Although none of these parameters has been shown to be a universal biomarker, evaluation in conjunction with classification systems such as that proposed by Govetto et al. has demonstrated greater accuracy in estimating postoperative functional potential [13, 19, 20].

In this context, most publications come from European, North American, and Asian cohorts, with a scarcity of data on Latin American populations, where demographic characteristics could influence clinical presentation and surgical outcomes.

Therefore, it is necessary to develop local studies that evaluate postoperative anatomical and functional outcomes in our setting, determining the prognostic value of structural biomarkers, applying state-of-the-art surgical techniques.

## Materials and methods

### Study design

This was a retrospective, analytical, longitudinal cohort study at the Fundación Oftalmológica Los Andes (FOLA) in Santiago, Chile, which included patients who underwent surgery between 1 January 2020 and 30 April 2025. Follow-up was conducted at baseline and then 1, 3, 6 and 12 months after surgery.

The design and writing of the study manuscript followed the STROBE guidelines [21].

### Objective

Determine the magnitude of change in best-corrected visual acuity (BCVA) at 12 months after ERM surgery based on preoperative OCT structural biomarkers, including Govetto’s classification (G2–G4), EIFL, EZ integrity, DRIL, COST line and central bouquet findings, in a cohort of eyes operated on over a 5-year period.

As secondary objectives, functional and anatomical responder were compared between idiopathic and secondary ERM, and between surgical techniques PPV alone vs. PPV combined with phacoemulsification (PPV/PHACO), and the preoperative prognostic value of these biomarkers on visual outcomes was estimated using adjusted multivariable models of these biomarkers on VA using adjusted multivariable models.

**Fig. 1:**
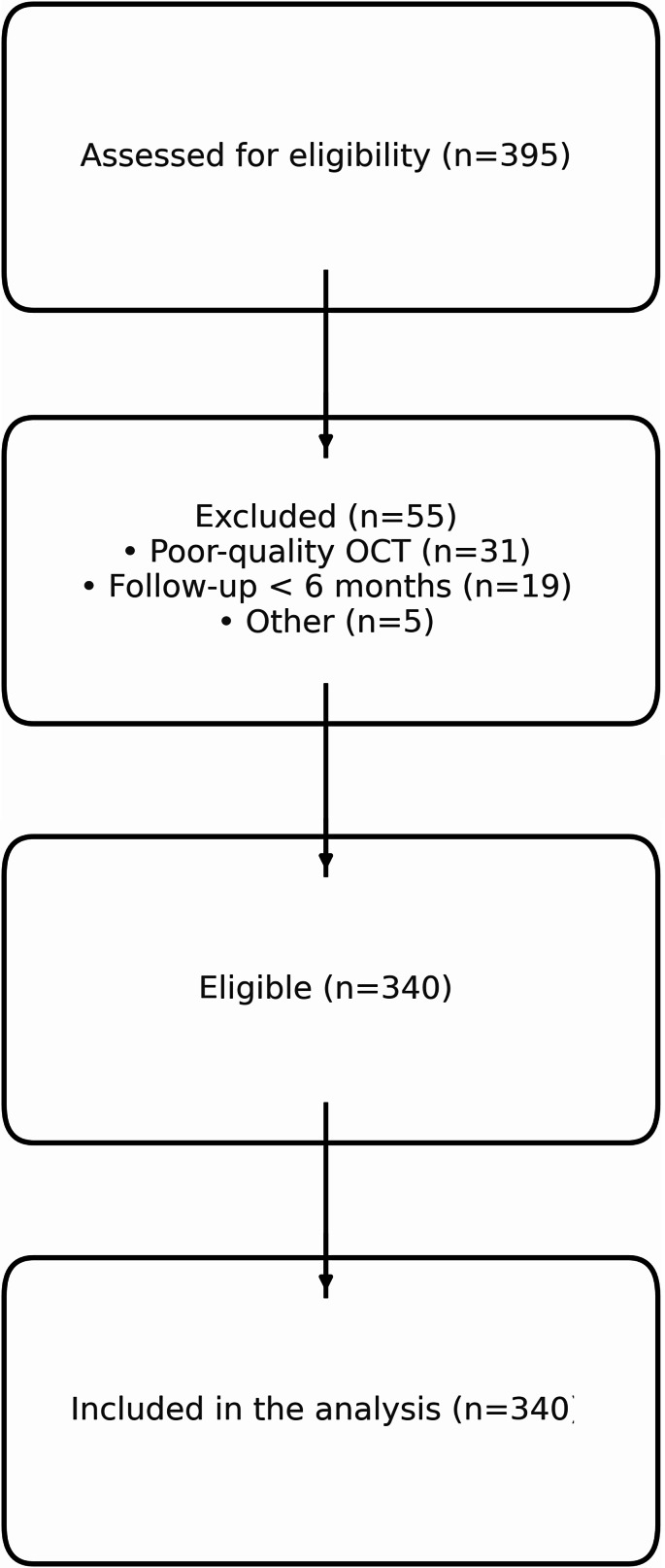
STROBE flow chart of cohort selection. OCT = optical coherence tomography

Inclusion and exclusion criteria are detailed in Table 1 and analysed using a selection diagram; Fig. 1.

**Table 1:** Inclusion and exclusion criteria

Category	Criterion
**Inclusion**	Phakic or pseudophakic eyes with idiopathic or secondary ERM (diabetic, post-cataract surgery, retinal vein occlusion, uveitis, trauma, previous retinal detachment).
	PPV surgery (23G/25G) with ERM peeling ± ILM peeling; subgroups according to PPV or PPV/PHACO technique and lens status.
	Good quality preoperative and postoperative Spectralis macular OCT (signal ≥ 7/10 or Q ≥ 25 dB), without critical artefacts.
	Follow-up ≥ 12 months with clinical evaluations and OCT at approximately 1, 3, 6 and 12 months.
	BCVA measured with ETDRS and converted to logMAR, recorded preoperatively and at 1, 3, 6 and 12 months.
	ERM in G2–4 at the time of surgery.
	For secondary ERM: controlled underlying disease
**Exclusion**	Active primary maculopathies unrelated to ERM that confound the outcome (active AMD, dystrophies).
	Untreated AMD or active proliferative retinopathy; extensive ischaemic RVO or with active neovascularisation; active uveitis.
	Progressive optic neuropathy (advanced glaucoma).
	VR surgery other than ERM in the previous 3 months.
	Poor-quality OCT, media opacities that prevent quality imaging.
	Loss of follow-up for less than 6 months or incomplete or missing critical data.
	ERM stage 1 at the time of surgery.

ERM = epiretinal membrane; PPV = pars plana vitrectomy; ILM = internal limiting membrane; VR: vitreoretinal; OCT = optical coherence tomography; BCVA = best-corrected visual acuity; ETDRS = Early Treatment Diabetic Retinopathy Study; logMAR = logarithm of the minimum angle of resolution; AMD = age-related macular degeneration; RVO = retinal vein occlusion

### Surgical technique

All eyes underwent PPV with ERM peeling, either as PPV alone or PPV/PHACO, according to routine clinical indication. The decision to perform combined PPV/PHACO was based on baseline lens status, the presence of visually significant cataract, and/or anticipated cataract progression that could affect postoperative visual rehabilitation; therefore, treatment allocation was not randomized. The decision to perform ILM peeling, as well as dye use and peel extent, was made by the operating surgeon according to intraoperative judgment and routine practice. Because ILM peeling details (including frequency, indication, dye use, and peel extent) were not uniformly documented across all cases in this retrospective cohort, ILM peeling was not included as a reliable exposure variable in the adjusted outcome analyses.

### Functional assessment

BCVA was measured using ETDRS charts under routine clinical conditions at each visit. When a standardized refraction was not performed at a given follow-up, measurements were obtained using the patient’s habitual correction and/or pinhole as clinically indicated. Very low vision categories were converted to logMAR using commonly used published conversions (counting fingers, hand motion, light perception and no light perception) to enable analysis on a continuous scale [22, 23].

The probability of being a “respond” was considered when the gain was greater than or equal to 0.2 logMAR (ΔlogMAR 12 m – baseline), which is related to a change of 2 lines of letters according to the Snellen chart and has been used as a threshold value in the literature to define significant functional changes in VA.

### OCT image acquisition

The images were obtained with SD-OCT Spectralis (Heidelberg Engineering, Germany), following a standardised and reproducible protocol between controls. Macular cubes with fields of 20°× 20° (6 × 6 mm) or 30°× 25° (8.8 × 7.3 mm) fields, with 49–97 B-scans. Automatic segmentation (ILM–BM) was used, and each volume was manually inspected to detect foveal segmentation errors.

### Qualitative analysis and operational definition of biomarkers

The analysis was performed by two ophthalmologists specialising in the retina, who were responsible for classifying the severity of ERM and identifying OCT biomarkers, tabulating data without access to the patient’s clinical information.

EIFL was defined using Govetto’s 2017 classification [13], recorded as a category from the apparent internal limit of the EIFL to the interface with the outer layers in the centre of the fovea.

G2 is defined as the loss of normal foveal depression, with some thickening of the outer nuclear layer (ONL), but without the formation of a continuous band of inner layer tissue in the fovea, and no EIFL. About G3, it is defined by the absence of foveal depression and the presence of a well-defined EIFL, with a continuous hypo/hyperreflective band corresponding to the inner nuclear layer (INL) plus the inner plexiform layer (IPL) crossing the foveal centre, although with the inner retinal layers still well defined, measurable between 50 and 250 μm centrally. Finally, G4 corresponds to advanced and thick ERM; EIFL is prominently present in the fovea, but with loss of normal laminar architecture, with internal layers appearing markedly disorganised. DRIL was defined as loss of distinguishable boundaries between the retinal nerve fibre layer (RNFL), the ganglion cell layer complex and the inner plexiform layer (GCIPL), the INL and the outer plexiform layer (OPL) in a 1 mm segment centred on the fovea; It was recorded according to severity as mild, moderate or severe, It was graded by the extent of DRIL within the central 1-mm segment (mild < 33%, moderate 33–66%, severe > 66% of the segment affected) identified on the horizontal axis of the central B-scan. EZ integrity was classified as intact or disrupted around the foveal centre, as a dichotomous variable for statistical analysis purposes. COST (cone outer segment tips; also referred to as the interdigitation zone, IZ) line was recorded as present, defined as a continuous line, or absent/discontinuous on the foveal axis. Central bouquet (external foveal complex) was recorded as the presence of the cotton ball sign [24], as a rounded hyperreflective lesion in the outer foveal layers, foveolar detachment and acquired vitelliform lesion, in the presence of subfoveal hyperreflective material with underlying shadow, also being evaluated dichotomously as present or absent. Of the quantitative parameters, the CMT is obtained in the central 1 mm ETDRS circle measured in microns (µm) at the foveal level.

### Statistical analysis

Continuous variables were described as mean with standard deviation (SD) or median (interquartile range, IQR), as appropriate, after verifying the normality of distributions using the Shapiro–Wilk test and the homogeneity of variances using Levene’s test. Categorical variables, such as sex, laterality, ERM aetiology, Govetto stage, presence of EIFL, DRIL, EZ, COST, and central bouquet, were summarised as absolute frequencies and percentages. For univariate comparisons of continuous variables between more than two groups, one-way ANOVA with Bonferroni-adjusted post hoc tests was used; when the assumptions of normality and/or variances were not met, the Kruskal–Wallis test with Dunn’s multiple comparisons and Benjamini–Hochberg multiplicity correction for multiple comparisons was applied. The comparison of proportions was performed using Pearson’s χ², employing Yates’ correction or Fisher’s exact test when the expected values were small.

The analysis of the dependent variable in our study was defined as the change in VA represented as Δ logMAR arithmetic difference between postoperative VA minus preoperative VA at 12 months; this was analysed with a paired Student’s t-test or, in the case of non-normality, with the Wilcoxon test for related samples. The difference in means was determined with 95% confidence intervals (CI) and Cohen’s dz effect size. Longitudinal VA follow-up at 1, 3, 6, and 12 months was analyzed using mixed-effects models including time, group, and time-by-group interaction terms. Fixed effects included time, aetiology, surgical technique, and biomarkers, in addition to the interaction terms time by group. All models were adjusted for clinical covariates and baseline BCVA in logMAR; the influence of the surgeon was evaluated in sensitivity analyses as a fixed factor or additional random term.

Differences between groups in functional response were estimated using an ANCOVA approach, analysing the postoperative logMAR value at 12 months as the dependent variable, with groups separated by aetiology or surgical technique as the factor and adjusted for baseline BCVA and preoperative biomarkers; this is equivalent to contrast of change under assumptions, reducing bias due to regression to the mean. The prognostic value of biomarkers was examined using multiple linear regression with Δ logMAR as the dependent variable and, exploratively, with logistic regression for the probability of being a “respond” with 95% CIs. The discrimination of EIFL, COST, EZ, and DRIL was evaluated with ROC curves, obtaining the area under the curve (AUC). For ordinal variables such as EIFL stage reversal, a proportional odds ratio (OR) model was used.

Within the time-to-event analysis, a time-to-response variable was constructed at 1, 3, 6, and 12 months, corresponding to the first follow-up at which response was achieved; non-responders were excluded at their last available follow-up. Overall Kaplan–Meier (KM) curves were estimated and stratified by Govetto stage, aetiology, and surgical technique, with log-rank comparison. Cox proportional hazards models were adjusted exploratorily; the proportional hazards assumption was verified with Schoenfeld residuals when appropriate.

For primary analyses, a two-sided p-value < 0.05 was considered statistically significant. For multiple post-hoc comparisons, adjusted p-values were used according to the correction methods specified above. All analyses were performed using jamovi v2.6 [25] and R v4.4 [26].

### A priori sample size calculation

This was based on the primary variable. The paired t-test formula was used to detect a conservative mean improvement of 0.10 logMAR, with a standard deviation of the difference of 0.25, bilateral significance level α = 0.05, and power 1 − β = 0.80.

## Results

### Demographics

The study cohort had a mean age of 73.4 ± 9.4 years with a median of 74 (IQR 69–80; range 17–93) and a proportion of women of 58.5% (199/340). Baseline BCVA was 0.54 ± 0.44 logMAR, median 0.45 (IQR 0.30–0.60; range − 0.16 to 3.00). Laterality was quantitatively similar, with 50.3% (171/340) in the right eye and 49.7% (169/340) in the left eye. The aetiology corresponded mostly to idiopathic ERM in 82.9% (282/340) versus secondary in 17.1% (58/340).

According to Govetto’s preoperative classification, G2 and G3 predominated with 48.8% and 42.9% respectively, and to a lesser extent G4 with 8.2%. The distribution of baseline OCT biomarkers and summarised categorical variables can be seen in Table 2.

**Table 2:** Baseline categorical characteristics of the cohort (*n* = 340 eyes)

Variable	Category	*n*	%
Sex	F	199	58.5
Laterality	OD	171	50.3
ERM aetiology	Idiopathic	282	82.9
	Secondary	58	17.1
Govetto	2	166	48.8
	3	146	42.9
	4	28	8.2
Surgical technique	PPV+PHACO	256	75.3
	PPV	84	24.7
EIFL	Present	155	45.6
	Absent	185	54.4
DRIL	No	209	61.5
	Mild (Mi)	54	15.9
	Moderate (Mo)	52	15.3
	Severe (S)	25	7.4
EZ	Disruption	198	58.2
	Integrity	142	41.8
COST	Disruption	204	60.0
	Full	136	40
Central bouquet	NO	163	48.1
	CBS	130	38.4
	VIT	47	13.9

ERM: epiretinal membrane; OD: right eye; PPV: pars plana vitrectomy; PHACO: phacoemulsification; DRIL: disorganisation of the retinal inner layers (none or absence, mild, moderate, severe); EZ: ellipsoid zone; COST: cone outer segment tips; Central bouquet: NO (no alterations), CBS: cotton ball sign, VIT: acquired vitelliform

### Overall functional analysis

**Fig. 2:**
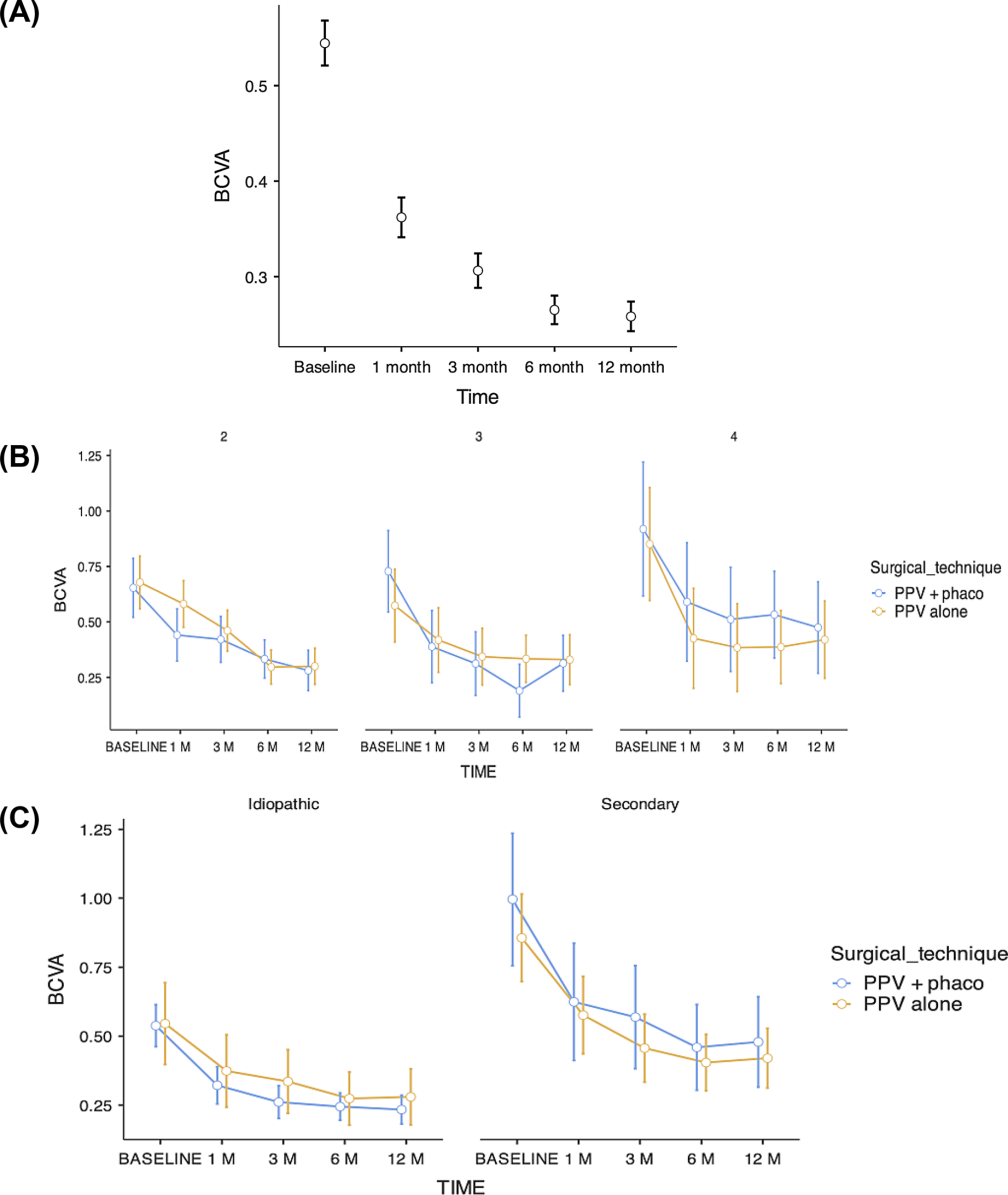
Evolution of BCVA after PPV by ERM. Evolution of BCVA in logMAR. **(A)** Overall curve at 12 months. **(B)** Curves stratified by Govetto stage and surgical technique. **(C)** Curves according to aetiology and technique

The primary objective of the study compared the change in BCVA, represented as ΔlogMAR = 12 m − baseline, showing an overall improvement with a mean of − 0.29 ± 0.42 and a median of − 0.23 (IQR − 0.42 to − 0.08; range − 2.37 to 1.08). Given that the distribution of differences did not meet Shapiro–Wilk normality *p* < 0.001, the Wilcoxon rank test was used, which confirmed a statistically significant improvement (W = 6422; *p* < 0.001). The effect size estimated as a biserial correlation of ranks was *r* = 0.78, consistent with a high clinical impact. In practical terms, the median of − 0.23 logMAR approximates a gain of approximately 11–12 ETDRS letters at 12 months. Figure 2 shows the behaviour of overall BCVA, and according to covariates, through longitudinal follow-up.

**Fig. 3:**
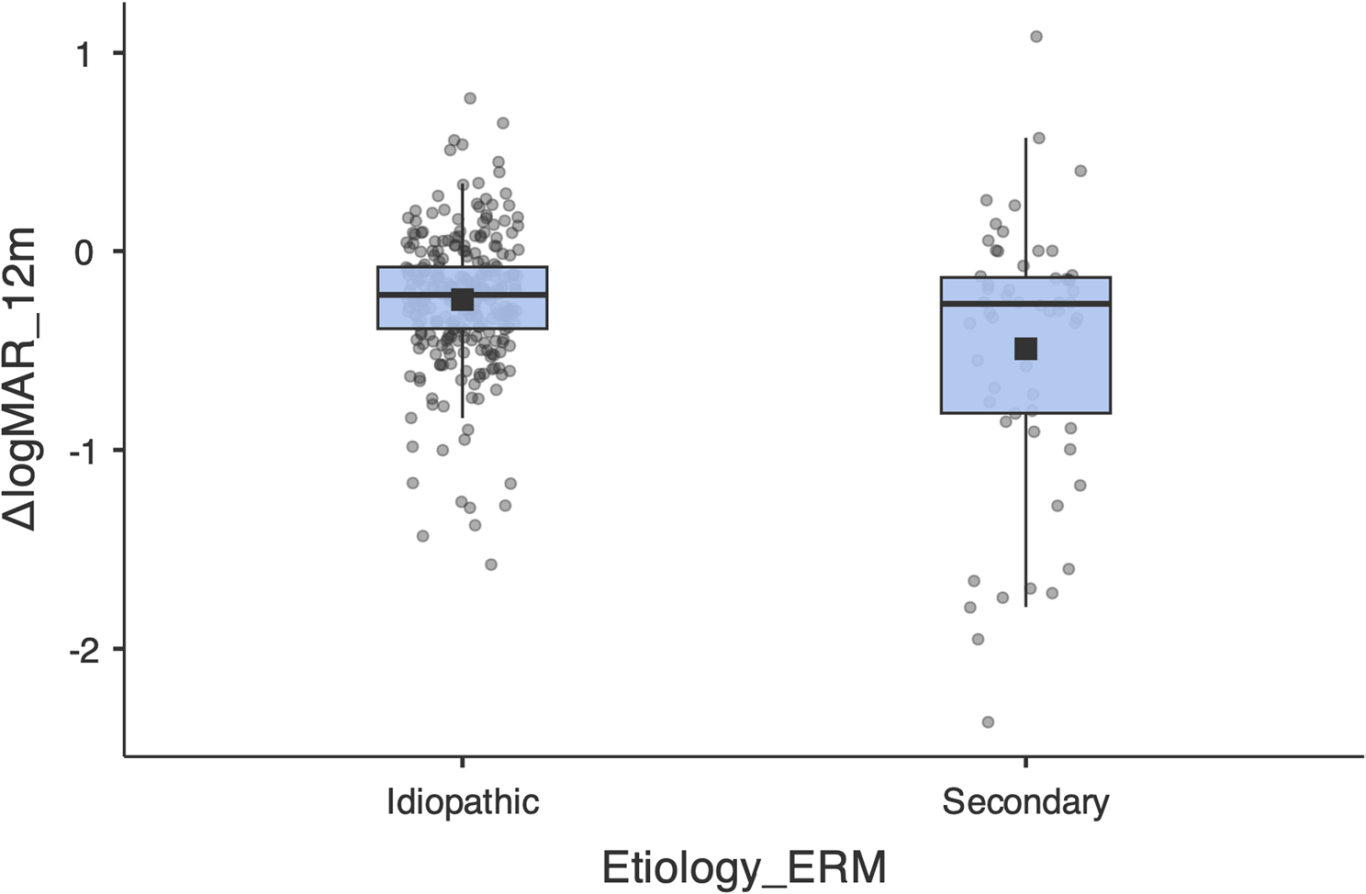
Change in BCVA at 12 months according to ERM aetiology. Boxplots of AVMC “ΔlogMAR = 12 m – baseline” by aetiology. The box shows IQR, the centre line the median, the whiskers 1.5×IQR; the dots are individual eyes and the black square the mean. Negative values indicate visual improvement. Mann–Whitney U = 6694.5; *p* = 0.0296); after ANCOVA adjustment (F = 1.88; *p* = 0.171)

When breaking down the visual results by aetiology, idiopathic ERMs (*n* = 282), representing 82.94%, when analysing BCVA, show a median of − 0.22 (IQR − 0.39 to − 0.08; mean − 0.24), while in secondary ERM (*n* = 58), representing 17.06%, it was − 0.27 (IQR − 0.82 to − 0.13; mean − 0.49). The Mann–Whitney comparison was statistically significant (U = 6694.5; *p* = 0.0296); Fig. 3. However, when adjusting for covariates in an ANCOVA model with ΔlogMAR at 12 months as the dependent variable and including baseline BCVA in logMAR, age, sex, aetiology and surgical technique, it is noteworthy that baseline BCVA was the most decisive predictor (β = −0.766; 95% CI − 0.834 to − 0.697; *p* < 0.001).

### Analysis according to surgical technique

**Fig. 4:**
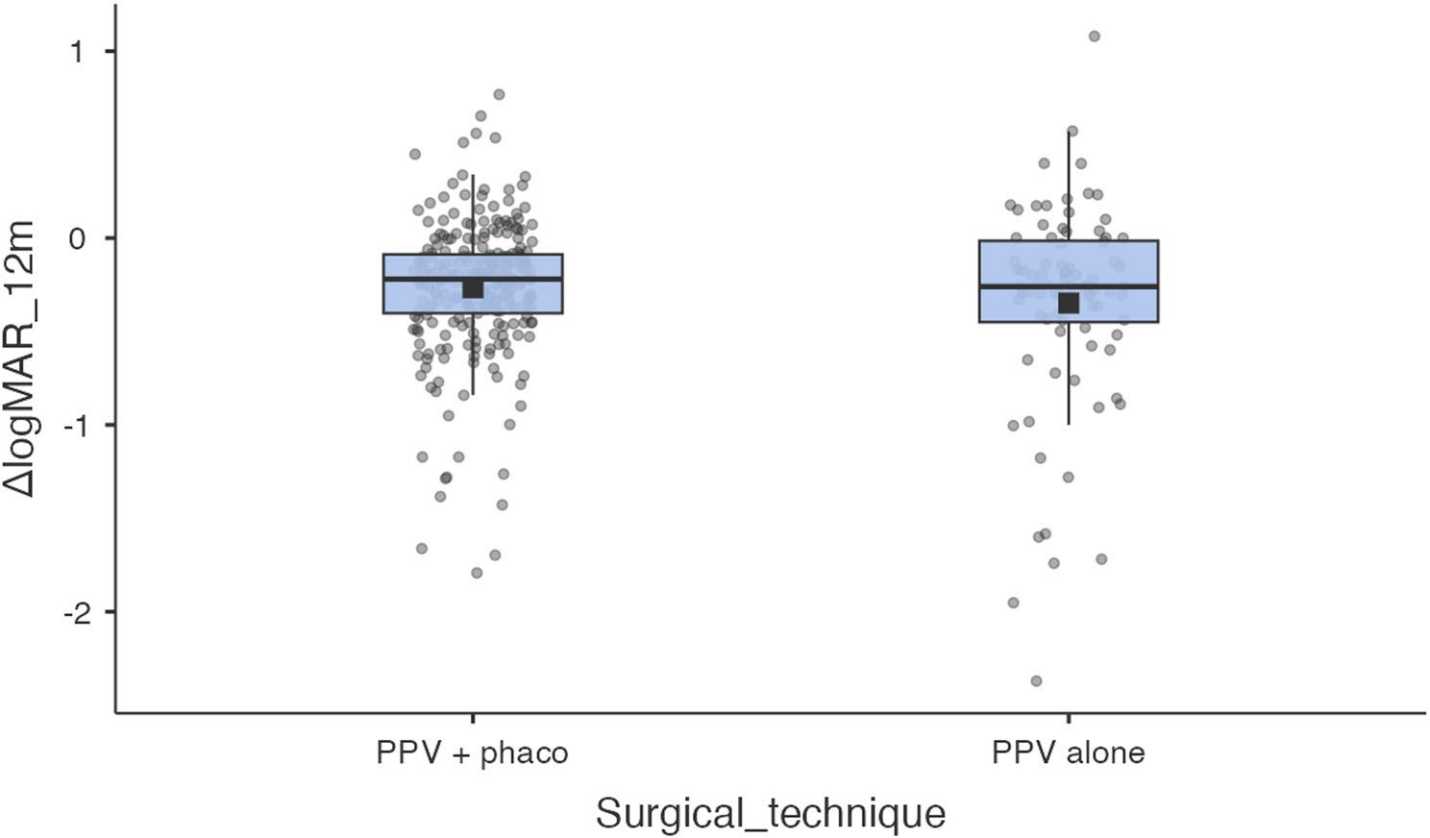
Change in BCVA at 12 months according to surgical technique. Boxplots of BCVA “ΔlogMAR = 12 m – baseline” according to surgical technique. The box shows the IQR, the centre line the median, the whiskers 1.5×IQR; the dots are individual eyes, and the black square is the mean. Mann–Whitney W = 10,244.5; *p* = 0.517). In adjusted models (β=+0.031 logMAR; 95% CI − 0.043 to 0.106; *p* = 0.408), and probability of being a responder (OR = 1.18; 95% CI 0.59–2.36; *p* = 0.625)

Of the 340 eyes, 256 (75.3%) underwent PPV+PHACO surgery and 84 (24.7%) underwent PPV surgery. Baseline BCVA was better in the PPV+PHACO group (mean 0.503 ± 0.360 logMAR; median 0.42; approximately 20/63 Snellen) than in the PPV alone group (mean 0.672 ± 0.600; median 0.45; approximately 20/94 Snellen); Fig. 4.

Functional improvement at 12 months was − 0.266 ± 0.362 in PPV+PHACO and − 0.348 ± 0.558 in PPV alone; the difference between techniques in ΔlogMAR was not significant (Mann–Whitney W = 10,244.5; *p* = 0.517). The proportion of responders was 53.9% in PPV+PHACO and 59.5% in PPV, with no significant differences in the comparison of proportions.

In the ANCOVA with 12-month BCVA as the adjusted dependent variable, the coefficient for PPV versus PPV+PHACO was β = +0.031 logMAR (95% CI − 0.043 to 0.106; *p* = 0.408). In the logistic regression for “responder” the adjusted OR for PPV versus PPV+PHACO was 1.18 (95% CI 0.59–2.36; *p* = 0.625). In both models, baseline VA remained the dominant predictor of functional outcome (*p* < 0.001).

### Analysis according to Govetto stage [13]

**Fig. 5:**
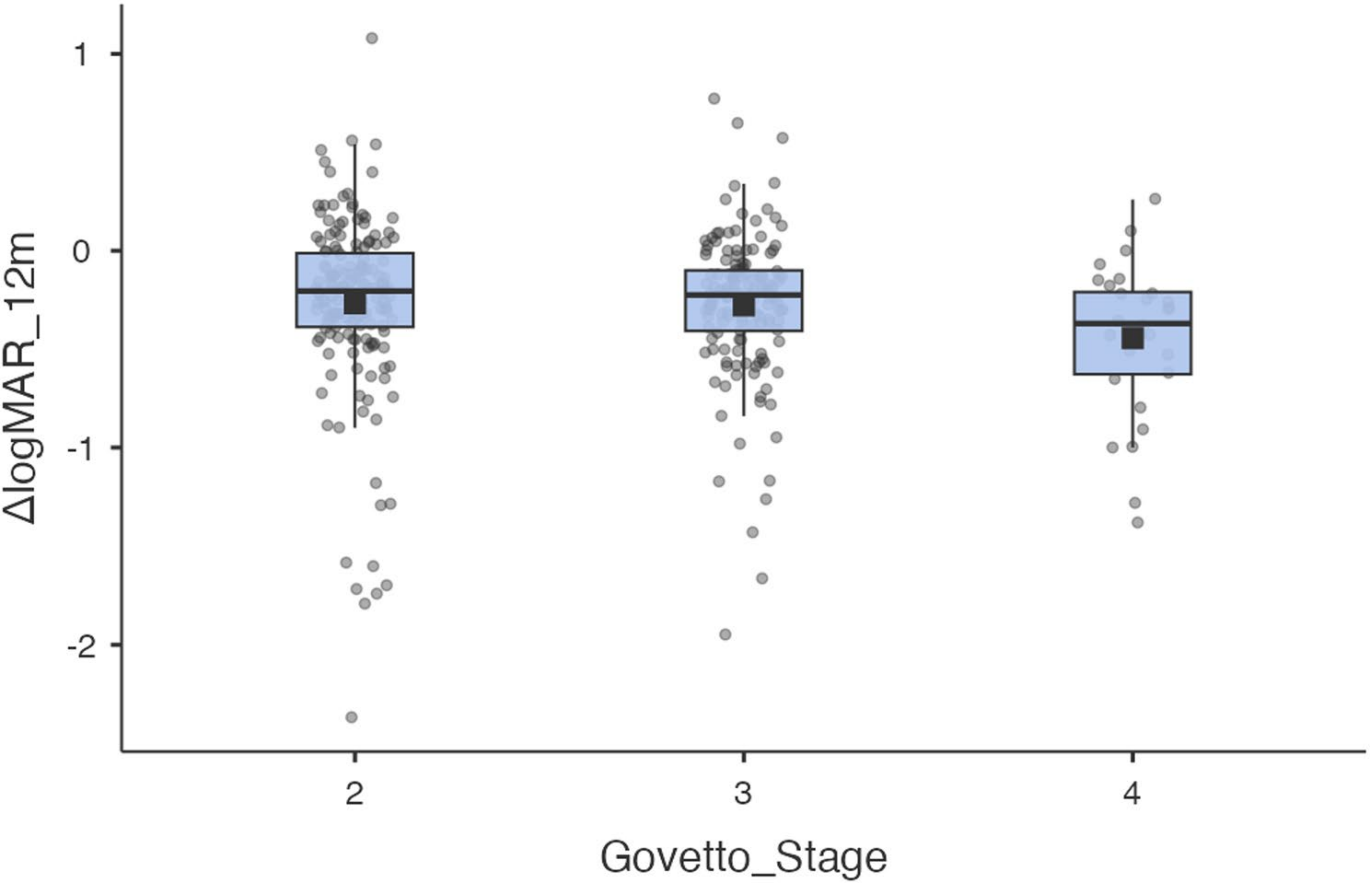
Visual change at 12 months according to Govetto stage. Boxplots of BCVA “ΔlogMAR = 12 m – baseline” stratified by Govetto: 2 (*n* = 166), 3 (*n* = 146) and 4 (*n* = 28). The centre line indicates the median, the box the IQR, the whiskers 1.5×IQR; black square the mean; grey dots = individual observations. Negative values represent greater visual gain. Comparisons between groups were evaluated using Kruskal–Wallis (H = 7.61; *p* = 0.0223; ε² = 0.0168)

According to the OCT classification, the medians for “ΔlogMAR 12 m – baseline” were, in G2: −0.2 (IQR − 0.380 to 0.000; *n* = 166), G3: −0.225 (− 0.408 to − 0.100; *n* = 146) and G4: −0.370 (− 0.628 to − 0.210; *n* = 28). This overall difference between stages was significant; Kruskal–Wallis H = 7.61; *p* = 0.0223; ε² = 0.0168 and, after correction for multiplicity according to the Benjamini–Hochberg method, a greater improvement was found in G4 compared to G2 and G3, with a p FDR = 0.0265 and p FDR = 0.0338, respectively, with no differences between G2 and 3, p FDR = 0.290; Fig. 5.

These findings are statistically significant and consistent with a response rate associated with structural severity: the higher the stage from G2 to G4, the greater the postoperative visual gain. However, when comparing the final BCVA, the more advanced the disease, the lower the final BCVA. Therefore, BCVA at each Govetto stage was consistent with the functional change. In G2, baseline BCVA was 0.519 ± 0.458 logMAR (approximately 20/66 Snellen), and BCVA at 12 months was 0.256 ± 0.280 logMAR (approximately 20/36 Snellen**)**, with a mean Δ of − 0.263 (SD 0.465). In G3, baseline BCVA was 0.553 ± 0.423 logMAR (approximately 20/71 Snellen), and at 12 months it was 0.268 ± 0.278 logMAR (approximately 20/37 Snellen), with a mean Δ of − 0.285 (SD 0.369). Finally, in G4, baseline BCVA was 0.933 ± 0.514 logMAR (approximately 20/171 Snellen), and at 12 months it was 0.345 ± 0.342 logMAR (approximately 20/44 Snellen), with a mean Δ of − 0.588 (SD 0.467); Table 3.

**Table 3:** Results by Govetto stage, preoperatively

Variable	G2 (*n* = 166)	G3 (*n* = 146)	G4 (*n* = 28)	*p*
BCVA, mean ± SD — Baseline	0.519 ± 0.458	0.553 ± 0.423	0.933 ± 0.514	*p* < 0.001
BCVA, mean ± SD — 12 months	0.256 ± 0.280	0.268 ± 0.278	0.345 ± 0.342	0.311¹
ΔlogMAR (12 m – baseline), mean ± SD	−0.263 ± 0.465	−0.285 ± 0.369	−0.588 ± 0.467	0.0223²

^1^ One-way ANOVA between stages; calculation based on means, SD and n per group

^2^ Kruskal–Wallis test between stages

### Analysis according to biomarkers

**Fig. 6:**
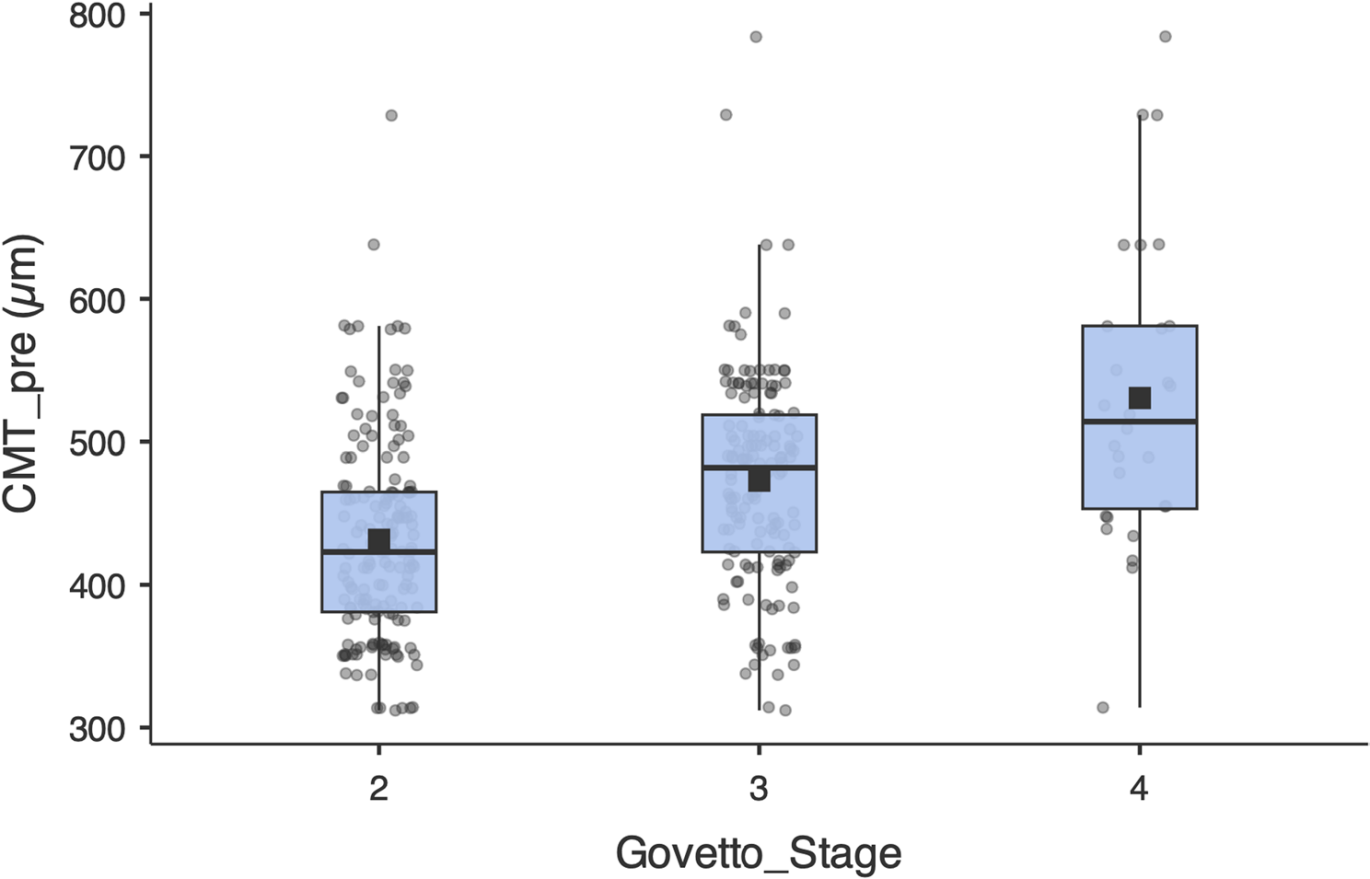
Baseline CMT according to Govetto stage. Boxplots represent the median and interquartile range (IQR); whiskers, 1.5×IQR; black square, mean; and dots, individual eyes. A progressive increase in CMT is observed with severity: medians 423 μm (E2), 482 μm (E3), and 514 μm (E4). Overall differences by Kruskal–Wallis: H = 41.80, *p* < 0.001

About structural biomarkers, the measured baseline CMT showed a mean of 457.5 ± 81.4 μm (median 449.5 μm; IQR 399.5–504). The association of baseline CMT with structural severity was highly significant. Increasing medians were observed between the different stages, 423 μm in G2, 482 μm in G3 and 514 μm in G4, with overall heterogeneity by Kruskal–Wallis (H = 41.80; *p* < 0.001) and a positive Spearman correlation of moderate magnitude between Govetto stage and CMT (rho = 0.346; *p* < 0.001), which confirms that macular thickening increases as the stage progresses. However, it did not correlate directly with final BCVA in the multivariate analysis (β = −0.09; *p* = 0.10); Fig. 6.

In the cohort, preoperative EIFL was present in 45.6% of eyes (155/340). As expected, EIFL was concentrated in more advanced Govetto stages, since it is intrinsically incorporated into the definition of stages 3 and 4. Therefore, this relationship should be interpreted as structural overlap within the staging system rather than as an independent association. EIFL was also associated with greater central macular thickness (rho = 0.272; *p* < 0.001).

In the 12-month analysis, resolved EIFL was associated with better BCVA in eyes that had this biomarker preoperatively and achieved longitudinal follow-up (*n* = 77; 69 with resolution and 8 with persistence). In the ANCOVA with 12-month BCVA as the multivariate-adjusted dependent variable, the effect of resolved EIFL was favourable but not significant (β= −0.161 logMAR; 95% CI − 0.720 to 0.397; *p* = 0.571). Consistently, in the binomial model for the probability of being a “responder” there was a trend toward a greater respond with resolved EIFL (OR 5.22; 95% CI 0.24–115.6), but it did not reach statistical significance (*p* = 0.296); Table 4.

**Table 4:** Prognostic value of biomarkers (preoperative) on response at 12 months

Preoperative biomarker	Adjusted association with “responder"¹	Univariate discrimination (ROC)²
COST	OR = 2.08 (95% CI 1.17–3.70); *p* = 0.013	AUC = 0.57
EIFL	NS*	AUC = 0.53
DRIL	NS*	AUC = 0.53
EZ	NS*	AUC = 0.52
Central bouquet	NS*	AUC = 0.56
Basal BCVA	Dominant predictor in all models; *p* < 0.001	—

^1^ Multivariate logistic regression; dependent variable “responder”: gain ≥ 0.2 logMAR at 12 months. Models adjusted for at least baseline AV, age, sex, aetiology, and surgical technique. OR > 1 indicates a higher probability of being a responder

^2^ Univariate ROC curves by preoperative biomarker. AUC interpreted as isolated discrimination of the marker

*NS: not significant after adjustment

At 12 months, baseline disruption of the COST line in OCT behaved as an independent predictor of visual response, particularly when continuity was recovered postoperatively. In the binary model for “responder,” having a disrupted baseline COST line was associated with approximately a 2-fold increased likelihood of responding (OR = 2.08; 95% CI 1.17–3.70; *p* = 0.013), after adjusting for baseline VA, clinical and anatomical covariates. Consistently, in the models with continuous VA, eyes that showed COST resolution achieved better VA at 12 months than those without recovery, with the effect remaining after multivariable adjustment.

**Fig. 7:**
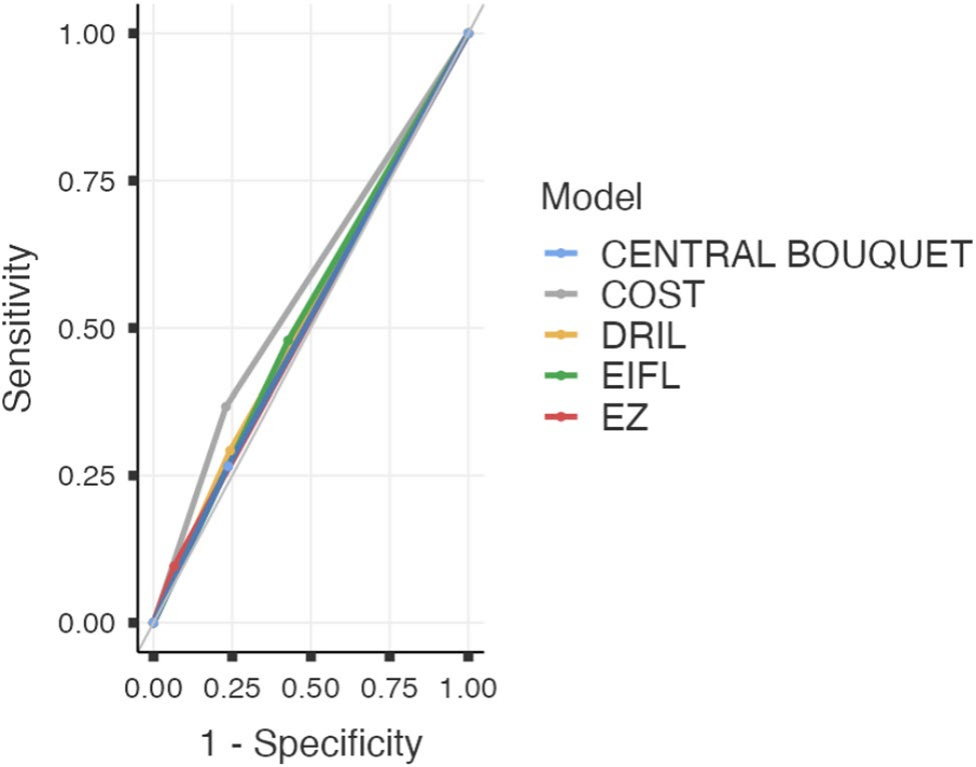
ROC curves of preoperative biomarkers to predict “responder”. Univariate ROC curves for the probability of being a “responder”. AUC (p-value): COST 0.568 (*p* = 0.005); EIFL 0.526 (*p* = 0.347); DRIL 0.525 (*p* = 0.308); EZ 0.515 (*p* = 0.310); CENTRAL BOUQUET 0.515 (*p* = 0.539). Only COST showed a modestly superior discriminatory capacity compared to random

EZ, DRIL, and central bouquet, when analysing pre- versus post-operative and prognostic value of “responder”. In the unadjusted analysis, postoperative restoration of the three biomarkers was associated with better VA at 12 months, while their persistence was associated with worse VA. However, when adjusted for baseline VA and clinical covariates, none showed an independent association in either the ANCOVA of VA at 12 months as dependent or in the binary regression for “responder” with *p* ≥ 0.05 in all cases. Their individual discriminatory capacity was limited, with AUC values of 0.515 for EZ, 0.525 for DRIL, and 0.56 for central bouquet; Fig. 7.

### Survival analysis

In the mixed linear models, a significant time effect was observed on BCVA, with early improvement that was maintained up to 12 months (*p* < 0.001). Baseline BCVA was 0.54 ± 0.44 logMAR (approximately 20/69 Snellen), and at 12 months it was approximately 0.26 logMAR (approximately 20/36 Snellen), consistent with the averages by technique; PPV+PHACO, 0.237 ± 0.242 logMAR (approximately 20/35 Snellen); PPV, 0.324 ± 0.377 logMAR (approximately 20/42 Snellen). The overall change at 12 months was − 0.29 ± 0.42 logMAR (median − 0.23; Wilcoxon *p* < 0.001).

In the entire cohort, the median time to response was close to 3 months. By severity according to Govetto stage, the medians were 3 months (G2, *n* = 166), 3 months (G3, *n* = 146) and 1 month (G4, *n* = 28). At 12 months, the probability of not responding was 30.1% (G2), 24.7% (G3) and 10.7% (G4), equivalent to cumulative response rates of 69.9%, 75.3% and 89.3%, respectively.

**Fig. 8:**
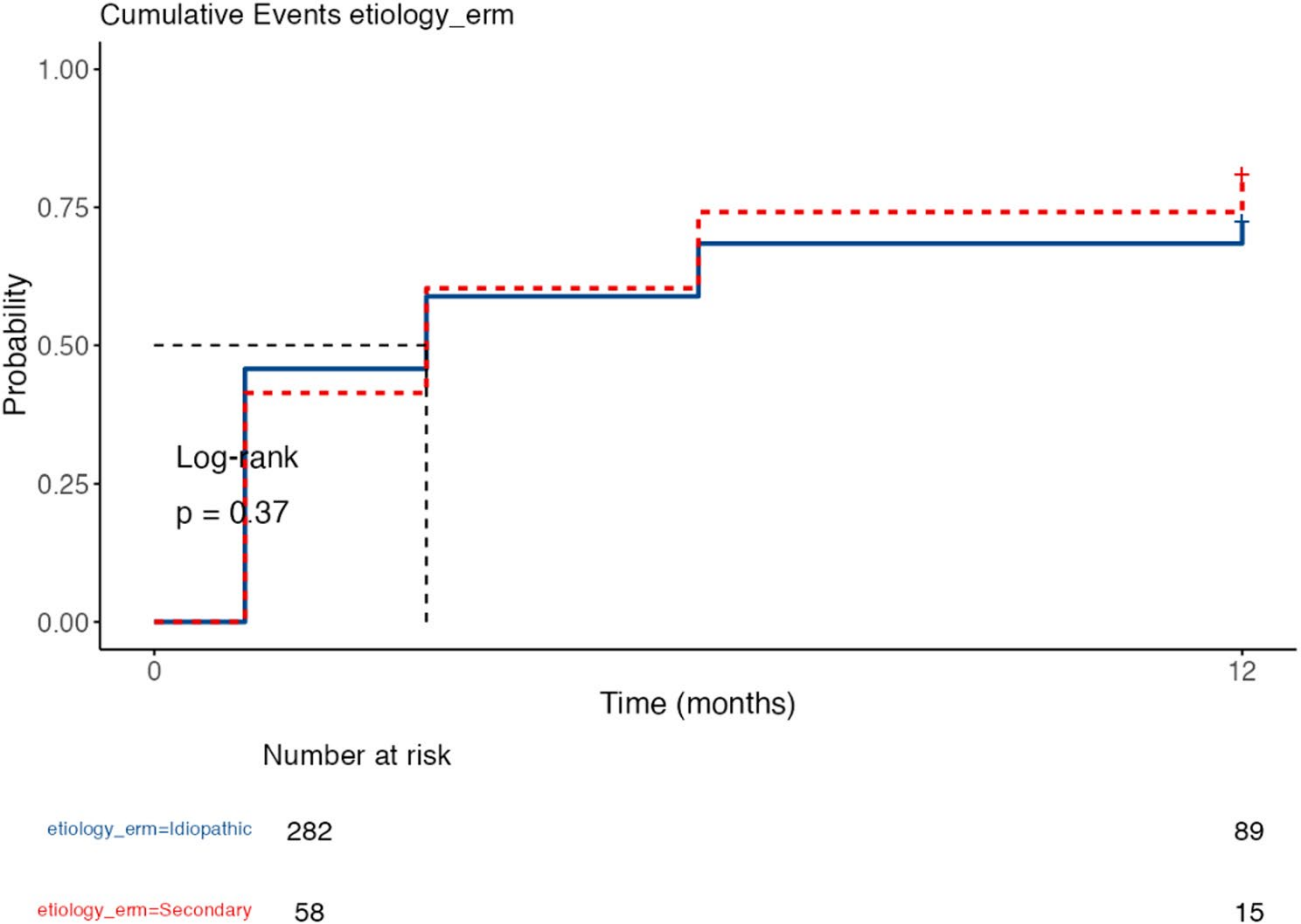
Kaplan–Meier of “time to responder” according to ERM aetiology. Cumulative incidence of being a responder (ΔBCVA ≥ 0.2 logMAR) according to aetiology. No significant differences were observed between idiopathic ERM (*n* = 282) and secondary ERM (*n* = 58) by log-rank test (*p* = 0.37). At 12 months, the cumulative incidence of responders was 72% in idiopathic ERM and 81% in secondary ERM. The Cox model confirmed the absence of significant differences between groups (HR = 1.13; *p* = 0.466)

**Fig. 9:**
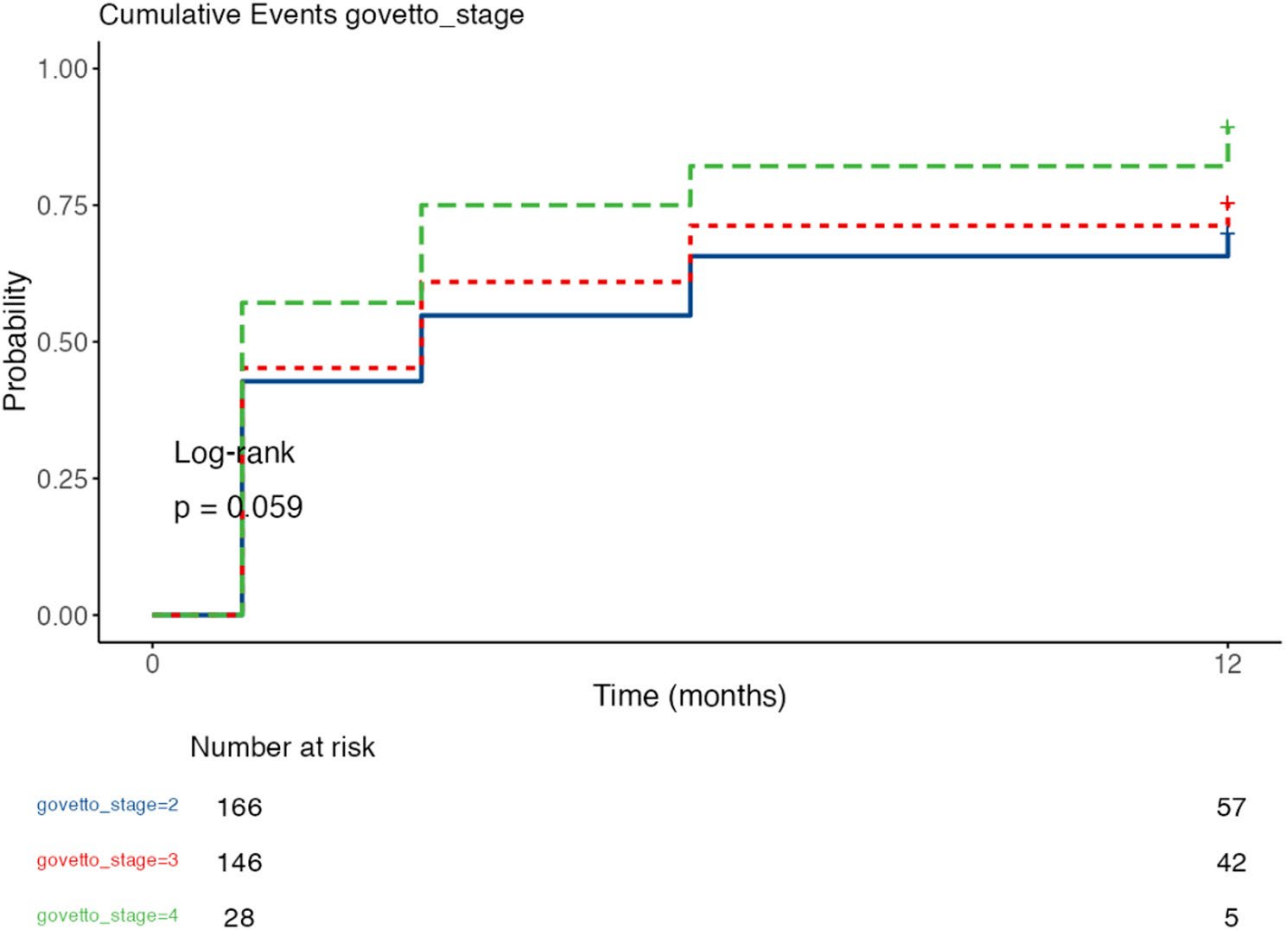
Kaplan–Meier curves of “time to responder” according to Govetto stage. Cumulative incidence of being a responder (ΔBCVA ≥ 0.2 logMAR) according to Govetto stage. There were no significant overall differences by log-rank test (*p* = 0.059). At 12 months, the cumulative responders rate was 69.9% in G2 (*n* = 166), 75.3% in G3 (*n* = 146) and 89.3% in G4 (*n* = 28). In the univariate Cox model, G4 showed a higher instantaneous responder rate vs. G2 (HR = 1.67; *p* = 0.021), with no differences for G3 (HR = 1.15; *p* = 0.302)

**Fig. 10:**
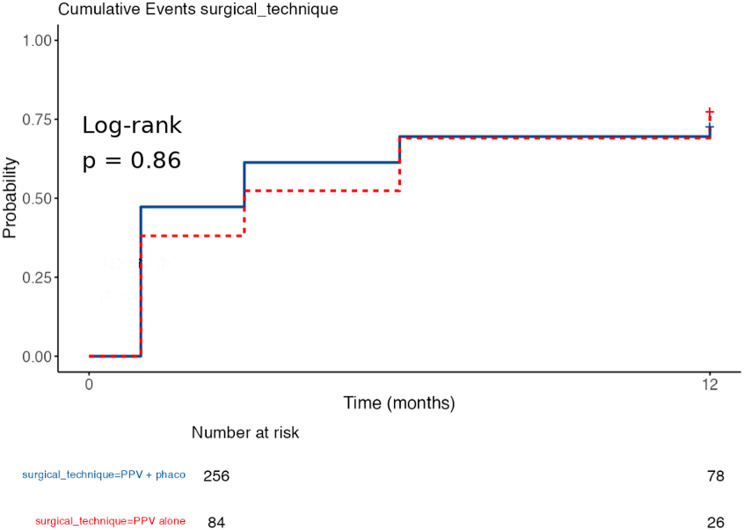
Kaplan–Meier curves of time to responder according to surgical technique. Cumulative incidence of being a responder (ΔBCVA ≥ 0.2 logMAR) according to surgical technique. No differences were observed between PPV+PHACO (*n* = 256) and PPV alone (*n* = 84) by log-rank test (*p* = 0.86). At 12 months, the cumulative incidence of responders was 73% and 77%, respectively. The Cox model confirmed the absence of significant differences between groups (HR = 0.97; 95% CI; *p* = 0.858).”

In the univariate Cox model, compared with G2, G4 had a higher response risk rate (HR = 1.67; *p* = 0.021), with no differences for G3 (HR = 1.15; *p* = 0.302). By aetiology, the medians were 3 versus 3 months and 12-month survival was 27.7% for the idiopathic group versus 19.0% for the secondary group, with no differences in the hazard ratio (HR = 1.13; *p* = 0.466). By technique, the medians were 3 months for the PPV+PHACO technique and 3 (3–6) months for PPV; 12-month survival rates were 27.3% vs. 22.6%, respectively; HR = 0.97; *p* = 0.858. The overall proportional hazards test was significant with *p* = 0.012; in the comparison by technique; consequently, these HRs should be interpreted with caution. (Figures 8, 9 and 10)

Finally, in the sensitivity analysis, the inclusion of the surgeon as a fixed factor or as a random term did not modify the results, whether considered as univariate or multivariate.

## Discussion

The main findings of this study were that ERM surgery was associated with significant visual improvement at 12 months, that baseline BCVA remained the strongest predictor of postoperative functional outcome, and that more advanced Govetto stages showed greater visual gain but lower final visual acuity ceilings. Among the evaluated OCT biomarkers, COST line status showed the most consistent prognostic signal, whereas EIFL reflected structural severity but was not an independent predictor in adjusted analyses. In addition, neither ERM aetiology nor surgical technique remained independently associated with final visual outcome after adjustment.

Our demographic results are consistent with series published in the literature, which highlight advanced age, with a mean of approximately 73 years, and a slight predominance of females. Meta-analyses indicate that advanced age and female sex are significant risk factors for developing ERM [1].

In the quantitative analysis, the data from our study variable confirm that PPV with ERM peeling produces a significant functional improvement, with a mean BCVA improved from 0.54 logMAR (approximately 20/70 Snellen) to 0.26 logMAR (approximately 20/40 Snellen) (median − 0.23), equivalent to a gain of around 11–12 ETDRS letters, corresponding to a gain of about 2 lines, with this improvement maintained for up to 12 months (Wilcoxon *p* < 0.001). This finding is consistent with previously published studies reporting clinically relevant improvements in VA after ERM surgery. De Clerck et al. reported an improvement in BCVA from 0.5 to 0.8 measured using Snellen charts up to one year after surgery in the idiopathic ERM group [27], and Kunavisarut et al. reported an increase in ETDRS of 51 to 65 letters at 6 months post-surgery [28]. In addition, other series detail in their results an improvement of around 70–77% of patients gaining at least two lines after membrane peeling [29] and Wong et al. reported a mean improvement of 0.31 logMAR (approximately 3 lines) with 83% of eyes improving ≥ 2 lines [30]. The effect size was large in our study (*r* = 0.78), reinforcing the clinical impact of surgery.

In the adjusted multivariate analysis, baseline BCVA stands out as the dominant predictor of postoperative gain.

We found that eyes with worse baseline or initial BCVA gained more, as reported by other authors [31, 32], who observed that 69.4% of patients improved their BCVA in the long term, with patients with worse initial BCVA gaining more; Wong et al. reported a direct correlation between pre- and post-operative BCVA, with quantitatively worse initial BCVA improving more after peeling [30, 33] .

About aetiology, we observed that secondary ERMs had worse preoperative vision and the greatest absolute gain on the logMAR scale, with a median of − 0.27 versus − 0.22 in idiopathic cases (*p* = 0.03). This coincides with studies by Kang et al., who found that test with secondary ERM had worse baseline BCVA but achieved greater postoperative visual improvement, albeit with a higher recurrence rate (20% versus 4.9%) [33].

Similarly, Norton et al. found that eyes with secondary ERM had lower preoperative VA and improved significantly after surgery but had a higher incidence of postoperative cystoid macular oedema [33, 34].

However, when we adjusted for covariates, including baseline VA, age, sex, and surgical technique, the aetiology lost independent significance (*p* = 0.171). The adjusted means of final gain were − 0.290 for idiopathic ERM and − 0.235 for secondary ERM, suggesting that the apparent advantage of secondary ERM is largely explained by their worse initial VA. This finding emphasises, as already noted, that initial visual acuity is key to the expected improvement.

In terms of surgical technique, we found no significant differences in visual outcome between PPV+PHACO and PPV alone, with ΔlogMAR = − 0.27 vs. − 0.35 (*p* = 0.517). Recent studies report similar findings. Dermer et al. compared combined versus deferred surgery in ERM and found no significant differences in VA gain or anatomical parameters between the two groups [35].

Our data are consistent with this conclusion and suggest that the combined approach does not alter adjusted visual improvement. Furthermore, the proportion of “responders” was comparable, with 54% for the combined surgery group versus 60% for PPV alone. Other groups have observed that combined surgery with an experienced anterior and posterior segment surgeon produces predictable refractive and visual outcomes like sequential surgeries [35, 36]. In the ANCOVA analysis and even in multivariable logistic regression, the surgical technique variable showed no independent effect on final VA (*p* > 0.4) or on the probability of responding (OR = 1.18; *p* = 0.625), reflecting that, after controlling for it, the attributable difference is marginal.

The Govetto classification, according to G2 to G4, was associated with the postoperative outcome. As other authors have shown, eyes in more advanced stages have worse preoperative VA but greater absolute gain. In our cohort, the medians obtained in ΔlogMAR were − 0.20, − 0.225, and − 0.370 in G2, G3, and G4, respectively. The Kruskal-Wallis test confirmed overall differences (*p* = 0.022) and adjusted post-hoc analysis indicated that G4 had superior gain compared to G2 and G3 (pFDR = 0.03). This pattern coincides with the observation by De Clerck et al. [27] in Retina 2025, where only eyes in G4 maintained significantly worse VA in the long term, demonstrating that structural severity predicts the outcome. Similarly, Govetto et al. [13] reported that, in OCT classification, BCVA progressively decreases from stage 1 to stage 4, emphasising that greater structural damage implies a worse prognosis. In summary, our findings suggest that, although advanced ERMs gain more letters, given their lower functional ceiling, their final BCVA remains lower. This highlights the importance of early detection; operating before very advanced stages could optimise long-term functional outcome.

Regarding preoperative OCT biomarkers, several have been linked to visual prognosis as the main functional variable. In our cohort, the presence of EIFL was observed in 45.6% and, as expected, was concentrated in more advanced Govetto stages because EIFL is structurally incorporated into the definition of stages 3 and 4. Therefore, this relationship should be interpreted as overlap within the staging system rather than as an independent association. In anatomical-functional terms, the biomarkers evaluated reflect the degree, type of traction and foveal microstructural damage induced by ERM. EIFL represents the ectopia of internal layers, mainly GCL and IPL, towards the centre of the fovea, secondary to tangential traction and the glial response of Müller cells; its presence is associated with loss of foveal depression, reduction of the foveal avascular zone (FAZ) and poorer visual function due to misalignment of the central photoreceptors. The literature establishes that EIFL is a marker of advanced damage. Govetto et al. [13] identified EIFL in approximately 33% of their cases and associated it with significant visual loss, incorporating it into their classification scheme. In line with this, Govetto found in 2019 that eyes with preoperative EIFL had worse pre- and postoperative VA (*p* < 0.001) [15]. In our analysis, the resolution of postoperative EIFL tended to be associated with better functional outcome (OR = 5.2 for responding ≥ 2 lines), but without reaching significance (*p* = 0.296), possibly due to the limited number of cases with this biomarker at the end of follow-up.

The integrity of the outer layers is key to sharp vision. The COST line reflects the coupling of the cone tips with the RPE; its baseline interruption usually indicates damage or misalignment of the outer segments and predicts more limited visual recovery if it persists. Classic and subsequent studies have shown that COST integrity correlates with postoperative VA in ERM and other maculopathies, while its prolonged loss is associated with worse outcomes [35].

Notably, we identified COST line disruption as the only preoperative biomarker with an independent effect on functional outcome. Shimozono et al. demonstrated that COST line integrity is a useful prognostic factor after ERM surgery [36].

Our findings reinforce this concept, as recovery from COST disruption at diagnosis almost doubled the odds of responding adequately after surgery (OR = 2.08; *p* = 0.013).

The EZ represents the integrity of the inner segments or mitochondrial bands of the cones; its postoperative restoration suggests metabolic and phototransduction recovery, but in our series, it had no independent effect after controlling for baseline VA and covariates. The literature is heterogeneous, as some studies find that interrupted EZ and DRIL are associated with worse VA and may have value in multivariate models, while others show modest discriminatory power in the short term. As for the inner layers, DRIL summarises the disorganisation of the boundaries between INL, IPL and GCL; its persistence translates into synaptic alteration and abnormal intraretinal conduction. Although in our study DRIL was not an independent predictor, specific ERM series have described associations with VA that may depend on stage, time of evolution and duration of EZ collapse. Similarly, alterations in the central foveal bouquet, which shows traction on the Müller fibre bundle and foveal cones, whose signs of central traction have been linked to poorer foveal morphology and metamorphopsia symptoms; their normalisation after peeling may accompany recovery, but their isolated prognostic power is limited [37, 38].

In fact, MacCumber et al. [36] reported that CMT and DRIL did not correlate significantly with visual gain, although the DRIL sample size was small, which is equivalent to that obtained in our experience.

After surgery, none of the postoperative structural biomarkers at one year showed a significant association with final adjusted VA.

The Kaplan–Meier curves show that the functional response already described as ≥ 0.2 logMAR occurs early, with a median of close to 3 months. Therefore, the expected improvement in the first 12 weeks and the surgical opportunity should consider that, although advanced stages respond earlier, they reach lower functional ceilings, favouring intervention before progression.

For this reason, our repeated measures models confirmed that most visual recovery occurs early. The study by Creuzot-Garcher et al. observed that VA improvement and foveal thickness reduction occur mainly in the first 6 months, with little additional gain beyond that point [39]. In fact, Romano et al. reported that the greatest increase in VA occurred 1 month post-surgery (*p* < 0.001) [40], and in our cohort we see a similar pattern of stabilisation after 1 to 3 months. This suggests that initial follow-up should be close and that VA remains at a plateau until one year.

This study provides a large cohort of patients in real-world practice, with serial follow-up and standardised anatomical characterisation. Our cohort reflects a Latin American tertiary referral setting, which may differ from US/European cohorts in referral patterns, timing of surgery, cataract status, and comorbidity burden. These factors may influence baseline disease severity and postoperative outcomes and should be considered when interpreting external generalizability. Variability in visual acuity assessment is a limitation of this study, as standardized refraction was not performed at every follow-up visit in all eyes and some measurements were obtained using habitual correction and/or pinhole. This may have introduced variability in postoperative functional outcomes. Variability in surgical technique is also a limitation of this study. Important intraoperative details, including the type of dye used and whether ILM peeling was performed, were not consistently documented across the cohort. This limited our ability to evaluate the independent effect of these surgical factors on postoperative outcomes.

However, the retrospective design limits generalisation and may leave room for confusion regarding indications; the dichotomous definition of “responder” simplifies the continuous response; interobserver variability of biomarkers was not quantified. Another limitation is the lack of quantitative assessment of metamorphopsia, a frequent and clinically relevant symptom in visually significant ERM. As BCVA does not fully capture symptom-based visual dysfunction, OCT biomarkers not independently associated with BCVA outcomes in our analyses may still have value for predicting functional symptoms such as metamorphopsia and aniseikonia. Future studies should incorporate standardized objective metamorphopsia measurements alongside structural OCT biomarkers and patient-reported outcomes. Nevertheless, the findings offer solid and clinically useful evidence.

## Conclusions

Finally, our results support international findings, where ERM surgery with PPV significantly improves BCVA, especially in patients with poorer initial acuity. The difference between idiopathic and secondary ERM disappears when adjusted for baseline AV, as does the choice of simultaneous versus deferred PHACO. The structural stage according to Govetto predicts the degree of gain, although not the final absolute VA value. In this cohort, EIFL was associated with greater structural disease severity, but it was not an independent predictor of visual outcome in adjusted analyses. Baseline BCVA remained the strongest determinant of postoperative functional outcome, while COST line status showed greater prognostic value among the evaluated OCT biomarkers. Overall, these results reinforce current guidelines and help to put our findings into clinical context in comparison with the international literature.

## Data Availability

No datasets were generated or analysed during the current study.

## Abbreviations

AMD: Age-related macular degeneration
BCVA: Best-corrected visual acuity
BM: Bruch’s membrane
CBS: Cotton ball sign
CI: Confidence interval
CMT: Central macular thickness
COST: Cone outer segment tips
DRIL: Disorganisation of the retinal inner layers
EZ: Ellipsoid zone
ETDRS: Early Treatment Diabetic Retinopathy Study
FC: Finger counting
GCIPL: Ganglion cell layer–inner plexiform layer complex
HM: Hand motion
ILM: Internal limiting membrane
INL: Inner nuclear layer
IPL: Inner plexiform layer
IQR: Interquartile range
logMAR: Logarithm of the minimum angle of resolution
LP: Light perception
NLP: No light perception
OCT: Optical coherence tomography
OD: Right eye
ONL: Outer nuclear layer
OPL: Outer plexiform layer
OR: Odds ratio
OS: Left eye
PHACO: Phacoemulsification
PPV: Pars plana vitrectomy
RNFL: Retinal nerve fibre layer
RVO: Retinal vein occlusion
SD: Standard deviation
SD-OCT: Spectral-domain optical coherence tomography
STROBE: Strengthening the Reporting of Observational Studies in Epidemiology
VR: Vitreoretinal
VIT: Acquired vitelliform (lesion)
